# Unveiling the Therapeutic Potential of *Atractylis aristata* Batt. Aqueous Extract: Anti‐inflammatory, Antioxidant, Antibacterial, Sedative Activities & Phytochemical Profiling

**DOI:** 10.1002/open.202500056

**Published:** 2025-04-17

**Authors:** Asma Abid, Nourelhouda Mekhadmi, Randa Mlik, Assia Bentahar, Kamilia Bireche, Bariza Frih, Walid Boussebaa, Aicha Mouane, Nezar Cherrada, Ana Sanches Silva, Messaouda Dekmouche, Lazhar Bechki, Khalid Mashay Al‐Anazi, Mohammad Abul Farah, Ahmad Ali

**Affiliations:** ^1^ Laboratory of Valorization and Promotion of Saharan Resources Faculty of Mathematics and Matter Sciences University of Ouargla Road of Ghardaia 30000 Ouargla Algeria; ^2^ Department of Biology Faculty of Nature and Life Sciences University of El Oued 39000 El Oued Algeria; ^3^ Laboratory of the Development and Technology of Saharan Resources (VTRS) Echahid Hamma Lakhdar El Oued University Algeria; ^4^ National Institute of Agronomic Research of Algeria INRAA, P. O. Box 299, Station of Adrar Adrar Algeria; ^5^ Laboratory of Phytotherapy Applied to Chronic Diseases SNV Faculty University of Setif 1 19000 Sétif Algeria; ^6^ Scientific and Technical Research Center in Physico-Chemical Analysis (CRAPC) Tipaza Algeria; ^7^ Laboratory of Biodiversity and Application of Biotechnology in Agriculture University of El Oued, El-Oued Algeria; ^8^ University of Coimbra Faculty of Pharmacy Coimbra 3000-548 Coimbra Portugal; ^9^ Centre for Animal Science Studies (CECA), ICETA 4099-002 Porto Portugal; ^10^ Associate Laboratory for Animal and Veterinary Sciences 1300-477 Lisbon Portugal; ^11^ Department of Zoology College of Science King Saud University Riyadh 11451 Saudi Arabia; ^12^ Department of Life Sciences University of Mumbai, Vidyanagari Mumbai 400098

**Keywords:** Atractylis aristata, Antibacterial, Anti-inflammatory, Antioxidant, Sedative

## Abstract

Medicinal plants possess the potential to yield bioactive compounds that offer significant health benefits; positioning them as valuable and promising sources for the development of innovative pharmaceutical products. This study aims to comprehensively assess the *in vitro* and *in vivo* pharmacological effects of the aqueous extract of the plant *Atractylis aristata* (AEAA) as well as assessments of its phytochemical composition. UPLC‐ESI‐MS/MS analysis of AEAA revealed a variety of bioactive compounds, including flavonoids and phenolic acids. In antioxidant assays, AEAA demonstrated considerable activity, with IC_50_ values of 0.269±0.05 mg/mL for DPPH scavenging and 0.0376±0.003 mg/mL for hydrogen peroxide radical inhibition. AEAA exhibited strong anti‐inflammatory activity *in vitro*, with an IC_50_ value of 2.563 mg/mL in the BSA denaturation test. *In vivo*, AEAA reduced carrageenan‐induced paw edema by 56.51 %, in comparison to an 83.58 % reduction with Ibuprofen®. Antibacterial testing showed AEAA′s broad‐spectrum activity, with the highest inhibition against *Bacillus subtilis* (34 mm zone of inhibition). Additionally, AEAA induced significant sedative effects, reducing locomotor activity by 48.98 %. These findings underscore the diverse pharmacological potential in addressing oxidative stress, inflammation, microbial infections, and anxiety of *A. aristata*, which can be attributed to its rich phytochemical profile.

## Graphical abstract

This study evaluates the pharmacological properties of the aqueous extract of *Atractylis aristata*, focusing on its antioxidant, anti‐inflammatory, antibacterial, and sedative activities. Phytochemical analysis identifies bioactive compounds, while *in vitro* and *in vivo* tests demonstrate its therapeutic potential in addressing oxidative stress, inflammation, microbial infections, and anxiety, offering a promising natural remedy for various health conditions.

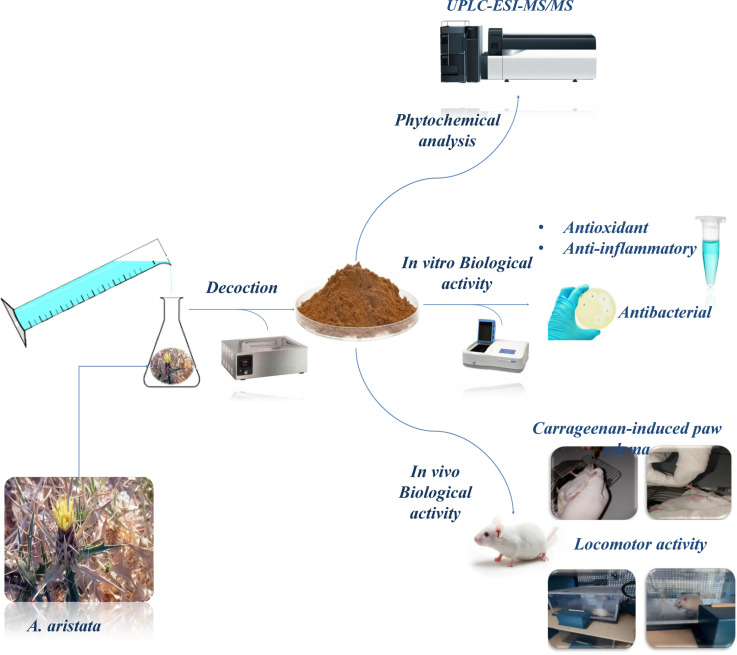

## 1 Introduction

Chronic inflammation is a persistent immune response that, while initially protective, can contribute to the development of numerous diseases, including cardiovascular disorders, autoimmune conditions, and metabolic syndromes. This inflammatory state is regulated by complex cellular pathways and mediators, such as cytokines and transcription factors, which are responsible for initiating and sustaining the inflammatory response. While pharmaceutical anti‐inflammatory drugs are commonly used to manage these conditions, they are often associated with adverse side effects and may not be suitable for long‐term use.^[1]^ Recently, natural anti‐inflammatory agents from plants and other sources have gained attention for their ability to modulate key inflammatory pathways like NF‐κB and JAK/STAT, without the side effects often seen with conventional drugs.^[2]^ Natural compounds help reduce inflammation by targeting cytokine production and immune cell function, offering potential benefits for autoimmune and inflammation‐related conditions.^[3]^

Oxidative stress, caused by an imbalance between reactive oxygen species (ROS) and the body‘s antioxidants, plays a key role in chronic diseases like Alzheimer's, cancer, and heart conditions. Natural antioxidants from plants, foods, and medicinal compounds help neutralize ROS, preventing cellular damage and maintaining balance. Their protective effects have made them a focus in disease prevention and treatment research, offering therapeutic benefits with fewer side effects than synthetic agents. These antioxidants work by scavenging free radicals and preserving mitochondrial function to reduce oxidative stress.^[4]^ Phytochemicals like flavonoids and polyphenols exhibit strong antioxidant activity, helping reduce the risk of chronic diseases in those with high fruit and vegetable intake. However, their role in diseases like cancer is complex, as they may interact with chemotherapy, highlighting the need for a deeper understanding of their mechanisms and applications.^[5]^

Sedation is essential in medical practice for managing anxiety, promoting relaxation, and facilitating procedures. Synthetic sedatives like propofol and sevoflurane are effective but do not fully mimic natural sleep patterns, with limited alignment to natural sleep stages. In contrast, dexmedetomidine more closely resembles non‐REM sleep stages 2 and 3.^[6]^ Interest in natural sedatives has grown, with plant‐derived compounds interacting with GABA receptors and serotonin transporters, offering effects similar to synthetic anxiolytics but with fewer side effects..^[7]^ Traditional plants like *Myristica fragrans* and *Hedychium spicatum* have long been used to treat insomnia and anxiety, supporting their use in modern therapies.^[8]^ Ethnobotanical research also highlights plants like *Valeriana officinalis* and *Lavandula angustifolia* for their sedative properties, further supporting their role in complementary medicine..^[9]^

The choice of using aerial plant parts and decoction as an extraction method is supported by both traditional practices and scientific evidence. Aerial parts, such as leaves and stems, are rich in bioactive compounds like flavonoids and phenols, contributing to the plant‘s medicinal properties, including antioxidative and anti‐inflammatory effects.^[10]^ These parts have been historically used in herbal remedies for conditions like diabetes and cancer.^[11]^ Decoction, a method that involves boiling plant material in water, effectively extracts these water‐soluble compounds, which enhances their bioavailability and therapeutic potential.^[10]^ Together, the selection of aerial parts and the use of decoction provide a powerful approach for optimizing the medicinal use of plants.

Phytochemical compounds in general are secondary metabolites produced by plants that play a significant role in defense mechanisms against pathogens, herbivores, and environmental stress. These compounds include alkaloids, flavonoids, terpenoids, tannins, phenolic acids, and saponins, among others, and are widely studied for their medicinal properties and potential health benefits. Atractylis genus has been used traditionally to treat ailments such as colds, dizziness, headaches, and inflammation. The plant *Atractylis aristata* Batt., is native to southern Algeria known for its traditional uses and pharmacological properties as antioxidant, anti‐inflammatory, sedative, and antidiabetic effects. Atractylis species such as *Atractylis flava* and *Atractylis gummifera* have shown antidiabetic activity, supporting the genus's potential in metabolic disorder management.[^12^, ^13^] The plant *A. aristata* has shown strong anti‐inflammatory effects and high ability to alleviate paw edema and BSA at low concentrations confirming its traditional uses to treat inflammation. In addition, the plant *A. aristata* has significantly reduced the locomotor activity in mice, exhibiting remarkable sedative activity and also promising antidiabetic properties.^[14]^ The pharmacological effect of *A. aristata* is significantly correlated with its phytochemical constituent's richness as acetylsalicylic acid, vanillin, gallic acid, quercetin, and ascorbic acid.[^14^, ^15^]

The genus Atractylis, traditionally utilized for treating conditions such as inflammation and pain, remains inadequately explored, with limited investigations into its pharmacological properties. Among the species within this genus, *Atractylis aristata* Batt. has been the subject of only a single published study regarding its medicinal effects. Known for its purported antioxidant, anti‐inflammatory, sedative, and antidiabetic activities, the full extent of its pharmacological profile has yet to be thoroughly investigated. This study aims to address this gap by evaluating the in vitro and in vivo biological activities of the aqueous extract of *Atractylis aristata*. Utilizing UPLC‐ESI‐MS/MS for phytochemical analysis, the research will identify the active compounds responsible for its therapeutic potential. Specifically, the study will assess its antioxidant, anti‐inflammatory, antibacterial, and sedative properties, thereby contributing to a deeper understanding of the genus Atractylis and its possible therapeutic applications.

## 2 Materials and Methods

### 2.1 Plant Material and Animals

#### 2.1.1 Plant Material

The species *Atractylis aristata* was gathered from its native habitat. The Algerian Sahara's Tamanrasset (22○47′ 13′′ N, 5○ 31′ 38′′ E) Terhanant region hosted the harvest during the winter in February 2019. The plant was identified by Prof. Reggani Abdelmalek Professor at University of Tamanrasset. The VPRS Laboratory herbarium received a plant specimen with the code 201902Tam/AtysAr. After harvesting, the fresh material was dried in a dark protected from sunlight and air.

#### 2.1.2 Animals

Mice of the Wistar variety, weighing between 18 and 20 g, were acquired from the Pasteur Institute of Algiers for the *in vivo* investigation. The mice are kept at 20–24 °C in polypropylene cages. The animals were given free access to food and water, and they were fed standard food (ONAB, Algeria). The animals were fasted for 16 hours prior to treatment in every experiment. This investigation was conducted at the SAIDAL Group's Research and Development Center in Algiers laboratories. The US rules and the globally recognized principles for the use and care of laboratory animals (NIH publication no. 85–23, amended in 1985) were followed in all animal experiments. The animal study protocol mentioned in this paper was approved by the Institu‐tional Review Board of El‐Oued university (protocol code 05/2023‐2024 and date of approval 08/02/2024).

### 2.2 Preparation of the Extract

The aqueous extract was prepared by the decoction of 50 g of the aerial parts plant *A. aristata* at temperature of 95 °C in distilled water for 1 h, after the filtration, the extract was frozen and then lyophilized using the freeze dryer to remove the water. Following drying, the aqueous extract of the plant *A. aristata* (AEAA) powder was conserved in airtight, moisture‐resistant containers and kept out of direct sunlight, heat, and moisture in a cold and dry location.

### 2.3 Quantitative Analysis

#### 2.3.1 Quantification of Total Phenolic Content (TPC)

Using the Folin‐Ciocalteu method with slight modifications by mixing 75 μL of AEAA, 275 μL of distilled water, and 150 μL of Folin‐Ciocalteu reagent (0.1 N) than the mixture was incubated for 3 minutes before adding 250 μL of 7.5 % sodium carbonate. After incubating for 30 minutes in the dark, a spectrophotometer measured the AEAA′s absorbance at 760 nm the results are reported in mg gallic Acid equivalents (GAE)/g dry matter.^[16]^

#### 2.3.2 Quantification of Total Flavonoids Content (TFC)

Total flavonoid content was calculated using Bahorun et al.′s (2004) technique. To utilize an aluminum chloride solution, by mixing 1 mL of extract with 1 mL of 2 % AlCl_3_. After 20 min, at 430 nm absorbance was measured and the results were expressed in mg quercetin equivalents (QE)/g dry matter.^[17]^

### 2.4 Qualitative Analysis Using UPLC‐ESI‐MS‐MS Analysis

The Shimadzu 8040, featuring ultra‐high sensitivity with the UFMS method and the binary bump Nexera XR LC‐20AD were utilized. Direct injection without columns: the standards have been optimized. The ESI criteria are as follows: When the ESI is turned on, the DL temperature is set to 250 °C, the nebulizing gas flow rate is 3.00 L/min, the heat block temperature is 400 °C, and the drying gas flow rate is 15 L/min. The CID gas pressure is 230 kPa. The mobile phases employed are water, 0.1 % formic acid, and methanol.^[18]^ At these conditions 5 μL of AEAA was injected and the detection of the phytochemical compounds was made by comparing the retention times with the injected standards both in the positive and negative modes.

### 2.5 In Vitro Biological Activity

#### 2.5.1 Antioxidant Activity

##### 2.5.1.1 DPPH Scavenging Activity

Cuendet et al. (1997) demonstrate the anti‐radical effect on 2,2‐dipheny‐ l‐1‐pycrylhydrazyl.^[19]^ The experiment proceeded as follows: 1250 μL of DPPH solution (0.004 % in methanol) and 50 μL of each test solution were mixed and then were left unsupervised at room temperature without illumination for 30 minutes; the BHT was used as standard in this test. Sample absorbance was measured at 517 nm and converted to a percentage. Radical scavenging activity was calculated using this formula:

(1) $$I\%={\frac{A_{0}-A_{A}}{A_{0}}}^{*}100\;\;$$

##### 2.5.1.2 Hydrogen Peroxide Radical Scavenging Activity

Fold‐dilution tested the AEAA′s hydrogen peroxide radical scavenging activity. 380 μL of AEAA at various concentrations was collected, and 62.5 μL of ammonium solution was added. It was mixed well before adding 17.5 μL of hydrogen peroxide. Solution incubation lasted 5 min after complete mixing. After incubation, 380 mL of ferroin was added and incubated for 10 min. At 230 nm, absorbance was measured. In the assay the vitamin C was used as positive control The hydrogen peroxides radical scavenging % was calculated using formula (1).^[20]^

##### 2.5.1.3 Iron Chelation Assay

Ferrozine is the most commonly used chelating agent in extract chelation testing. Ferrozine complexes with free iron in the medium are used to generate bright purple Ferrozine. The AEAA′s ability to bind iron was tested by stopping the formation of the complex Fe^2+^‐ferrozine, following the steps outlined by Le et al. (2007).^[21]^ Add 50 μL of FeCl_3_ (0.6 mM) and 450 μL of methanol to 250 μL of AEAA quantities, mix, and let sit for 10 minutes at room temperature. After adding 50 μL of ferrozine, the absorbance was measured at 562 nm after 10 min, compared to the control EDTA, which contained all reactants except the AEAA.^[22]^

##### 2.5.1.4 Ferric Thiocyanate (FTC) Method

AEAA prevented linoleic acid peroxidation using a modified ferric thiocyanate method. The FTC method detected lipid peroxidation‘s first peroxide. Red ferric chloride (FeCl₃) is formed from peroxide and FeCl₂. This reduces peroxide by increasing antioxidant activity. Mix 0.5 mL AEAA with 2.5 mL of 0.02 M linoleic acid emulsion at pH 7.0 and 2 mL of 0.2 M phosphate buffer. The linoleic acid emulsion contained 50 ml of phosphate buffer, 0.2804 grams of fatty acid, and 0.2804 grams of Tween 20. The reaction mixture was incubated for five days at 37 °C. 0.1 ml of the reaction mixture was added to 4.7 ml of 75 % ethanol, 30 % ammonium thiocyanate, and 0.02 M ferrous chloride in 3.5 % HCl every 24 hours. The reaction mixture‘s absorbance was measured at 500 nm three minutes after ferrous chloride was added and repeated.^[23]^ The FTC delineated antioxidant activity through % inhibition, represented by the following formula:
(2)

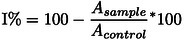




***A***_***Contro**l*_: is the absorbance of the control reaction with simply linoleic acid emulsion and sodium phosphate buffer, while ***A***_***Sample***_: is the absorbance with AEAA or standard Vitamin C or BHT.

##### 2.5.1.5 Thiobarbituric Acid (TBA) Method

The TBA assay employs spectrophotometry to measure the pink color shift that results from two TBA molecules reacting with an MDA molecule at low pH and high temperature (100 °C), thereby inferring the production of MDA from linoleic acid peroxide. Similar sample preparation was done as in FTC. 1 mL of sample solution was mixed with 2 mL of 20 % trichloroacetic acid and 2 mL of thiobarbituric acid. Boil the mixture for 10 minutes. The sample is centrifuged at 3000 rpm for 20 minutes, and after cooling, the supernatant absorbance is measured at 532 nm to determine antioxidant activity on the last day of the FTC experiment. The TBA quantified antioxidant activity via % inhibition, as indicated by formula (2).^[24]^

Where: ***A***_***Control***_: is the absorbance of the control (without the sample). ***A***_***Sample***_: is the absorbance with AEAA or standard Vitamin C or BHT.

#### 2.5.2 In Vitro Anti‐Inflammatory Activity

The *in vitro* anti‐inflammatory activity of AEAA′s was assessed using denaturation of bovine serum albumin (BSA) and denaturation of egg albumin (EA) methods.

##### 2.5.2.1 Denaturation of Bovine Serum Albumin (BSA)

Different concentrations of AEAA were prepared in Tris‐HCl (0.02 M, pH 6.6). Each concentration was combined with a 2 % bovine albumin aqueous solution in equal proportions. The reaction mixture was incubated at 37 °C for 15 min and then heated at 72 °C for 5 min. After cooling, the absorbance was measured at 650 nm.^[25]^ The Aspirin® was used as a positive control and the albumin denaturation inhibition percentage of AEAA and Aspirin® was calculated using equation (3) and the results were expressed by IC_50_ values.

(3) $$I\%={\frac{A_{0}-A_{A}}{A_{0}}}^{*}100\;$$

##### 2.5.2.2 Denaturation of Egg Albumin (EA)

AEAA was tested for its anti‐inflammatory activity in fresh eggs utilizing Bouaziz et al.′s protein denaturation method. Fresh eggs were properly washed and broken to separate whites and yolks. Measure the egg white volume and add it to a previously prepared Tris‐HCl (20 mM, pH=6.8) solution to dilute it (1/100, v/v). After shaking gently for 10 minutes, the solution was filtered. 2 ml of this solution was added to test tubes with varied extract or Aspirin® concentrations. After incubation at 74 °C for 30 minutes the absorbance was measured at 650 nm.^[26]^ Protein degradation inhibition was calculated using formula (3) and the results were expressed by IC_50_ values.

### 2.6 Antibacterial Activity

Pasteur Institute of Algeria microorganisms were used in this investigation. Two Gram‐positive species were tested: *Staphylococcus aureus* ATCC 2592 and *Bacillus subtilis* ATCC 6633. Three Gram‐negative species were also tested: *Pseudomonas aeruginosa* ATCC 27853, *Escherichia coli* ATCC 25922, and *Klebsiella pneumoniae* ATCC 70603. Bacteria with an optical density of 0.08–1 at 625 nm were grown overnight in a physiological saline (0.8 % NaCl) broth culture. Müller‐Hinton agar (MH agar) was solidified and dried on Petri dishes before inoculation. The agar plates inoculated with bacteria were overlaid with 6 mm sterile discs and treated with 100 μl of both diluted and undiluted extracts (25, 50, and 100 v/v DMSO: Dimethylsulfoxide 10 %). The positive control was a solution with 15 μg of Gentamicin, while the negative control was DMSO. Each test was repeated three times and averaged. Petri plates were incubated aerobically at 37 °C for 18–24 hours. In these investigations the extract sensitivity was measured by inhibition zone diameter following incubation.^[22]^ 

### 2.7 In Vivo Biological Activities

#### 2.7.1 In Vivo Acute Toxicity Study

The AEAA acute oral toxicity study used the oral route limit of 2000 mg/kg body weight from the Test Guidelines on Acute Oral Toxicity (OECD guideline No. 423, 2002). Six female Albino mice were selected, weighed, and given 2000 mg/kg body weight AEAA. Following a 16‐hour fast, each mouse received 0.5 mL AEAA via stomach tube.^[27]^

#### 2.7.2 In Vivo Anti‐Inflammatory Activity

AEAA was tested for anti‐inflammatory efficacy in adult mice utilizing Carrageenan‐induced paw edema. Mice were randomly assigned to 3 homogenous groups of 5–6.^[28]^ In this assay the test group administrated 0.5 mL of 2000 mg/Kg AEAA, while the control group and the reference group administrated 0.5 mL of physiological water and Ibuprofen®, respectively. 30 minutes after treatment, each mouse received an injection of 0.025 mL of carrageenan (1 %) under the left hind paw pad. Without therapy, the right hind paw was a control. The formulas (4) and (5) were used to calculate the percentage increase in paw weights (Edema %) and the Edema Reduction Percentage (ERP %) in treated mice compared to controls.
(4)

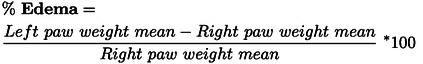

(5) $$ERP\%=\;{\frac{\%\;control\;edema\;mean-\%\;edema\;test\;mean}{\%\;control\;edema\;mean}}^{*}100\;\;$$

#### 2.7.3 In Vivo Sedative Activity

The locomotor activity of AEAA was evaluated using the actophotometer technique.^[29]^ The animals were categorized into three groups, each including five to six individuals. In this assay the test group administrated 0.5 mL of 2000 mg/Kg AE AA, while the control group and the reference group administrated 0.5 mL of physiological water and Haloperidol®, respectively. Thirty minutes after, each mouse was positioned in the actophotometer for thirty minutes. A light beam is interrupted by the animal‘s movement and strikes a photocell, which registers and exhibits a digital count in the corresponding totalizer. The outcomes of the sedative action were quantified by the Movement Reduction Percentage (MRP), obtained using formula 6.

(6) $$MRP\;I\%=\;{\frac{Control\;movment\;mean-Test\;movment\;mean\;}{Control\;movment\;mean}}^{*}100\;$$

### 2.8 Statistical Analysis

Triplicate assays were done and the results expressed by mean ± SD. Statistical analysis were executed with R 4.4.2 software and Microsoft Excel 2016. Significance was defined at p <0.05. The one‐way analysis of variance (ANOVA) was used to compare the means between sample and standards. Network graph analysis and Person heatmap correlations were applies to examine the relationships between antioxidant evaluation assays. For comparison between the antibacterial potential of sample and doses, Two‐way analysis of variance (ANOVA) was validated. Principal Component Analysis (PCA) was performed using the mean values of triplicate to correlate the results of tested biological activities with the TPC and TFC amounts.

## 3 Results

### 3.1 UPLC‐ESI‐MS‐MS Analysis

**Figure 1:**
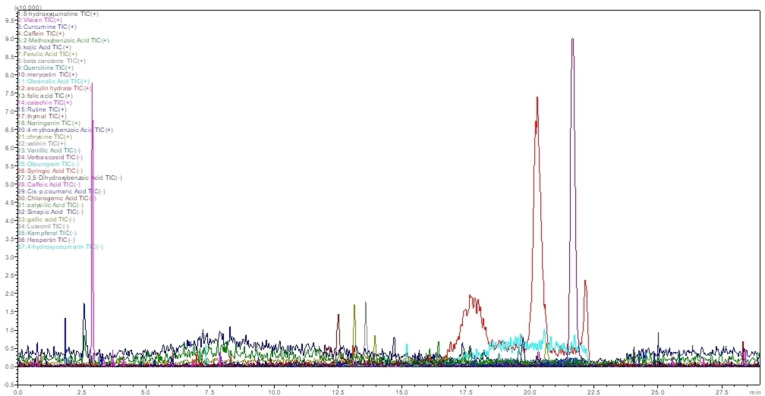
UPLC‐ESI‐MS‐MS Chromatogram of AEAA.

**Figure 2:**
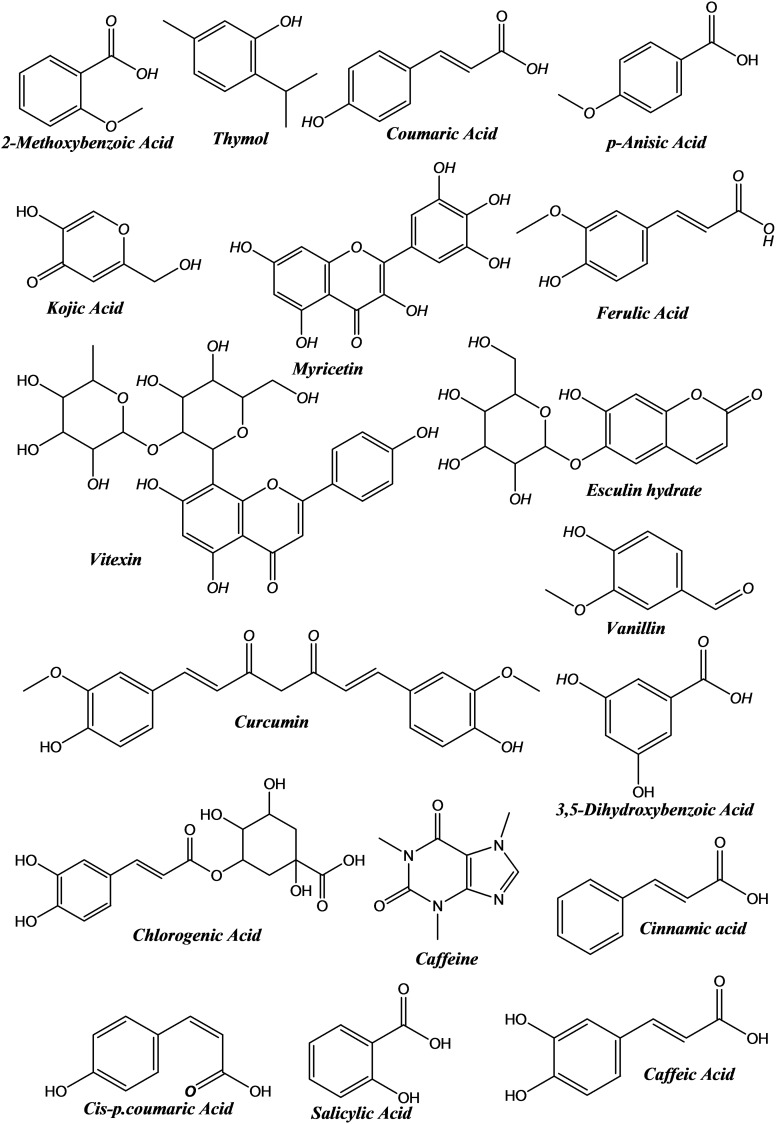
Chemical structures of the compounds detected in the AEAA by UPLC‐ESI‐MS‐MS analysis.

Table 1 summarized the results of UPLC‐ESI‐MS‐MS analysis of AEAA by comparing the retention times with the injected standards both in the positive and negative modes. The UPLC‐ESI‐MS study of the aqueous extract from *Atractylis aristata* revealed the presence of variety bioactive chemicals as shown in the Figure 1, categorized mostly into phenolic acids, flavonoids, and other significant secondary metabolites. Identified key phenolic acids comprise 2‐methoxybenzoic acid, p‐Anisic Acid, coumaric acid, ferulic acid, cinnamic acid, and chlorogenic acid and two flavonoids, including vitexin and myricetin. Furthermore, esculin hydrate, a derivative of coumarin, was identified the AEAA and the thymol, a monoterpene phenol, other compounds are detected in AEAA such as: curcumin, caffeine and kojic acid. This detailed chemical profile highlights the abundance of bioactive chemicals in *Atractylis aristata*. The chemical structures of the identified compounds are drawn in the Figure 2.

**Table 1:** Phytochemical constituents detected in AEAA via UPLC‐ESI‐MS‐MS analysis.

Compound	Rt (min)	Molecular Formula	Molecular Weight	Ion mode	Transtion m/z	Phytochemical classes
Coumaric Acid	2.575	C_9_H_8_O_3_	164.16	Positive	165.1000>101.2000	Phenolic Acid
p‐Anisic Acid	2.576	C_8_H_8_O_3_	152.15	Positive	153.0500>70.7500	Phenolic Acid
2‐Methoxybenzoic Acid	2.583	C_8_H_8_O_3_	152.15	Positive	153.0500>135.0500	Phenolic Acid
Chlorogenic Acid	11.279	C_16_H_18_O_9_	354.31	Negative	353.0500>191.1000	Phenolic Acid
Thymol	12.231	C_10_H_14_O	150.22	Positive	151.7500>88.1000	Terpenoid
Caffeine	12.477	C_8_H_10_N_4_O_2_	194.19	Positive	195.1000>137.9000	Alkaloid
Caffeic Acid	12.573	C_9_H_8_O_4_	180.16	Negative	–	Phenolic Acid
3,5‐Dihydroxybenzoic Acid	12.638	C_7_H_6_O_4_	154.12	Negative	153.1000>109.1000	Phenolic Acid
Curcumin	12.809	C_21_H_20_O_6_	368.4	Positive	369.0000>145.0500	Polyphenol
Vallinin	13.582	C_8_H_8_O_3_	152.15	Positive	153.1000>65.1500	Phenolic aldehyde
Vitexin	13.649	C_21_H_20_O_10_	432.4	Positive	433.0000>283.0000	Flavonoid
Cis‐p.coumaric Acid	13.807	C_9_H_8_O_3_	164.16	Negative	163.1500>119.1500	Phenolic Acid
Ferulic Acid	13.925	C_10_H_10_O_4_	194.18	Positive	194.9000>177.1500	Phenolic Acid
Kojic Acid	14.654	C_6_H_6_O_4_	142.11	Positive	–	Phenolic compound
Salysilic Acid	15.491	C_7_H_6_O_3_	138.12	Negative	137.1000>93.1500	Phenolic Acid
Myricetin	21.661	C_15_H_10_O_8_	318.23	Positive	336.2500>46.1500	Flavonoid
Cinnamic acid	21.664	C_9_H_8_O_2_	148.15	Positive	149.0500>84.7500	Phenolic Acid
Esculin hydrate	22.205	C_15_H_18_O_10_	358.3	Positive	359.1000>295.1500	Coumarin
Rt: Retention time						

### 3.2 Total Phenolic Content and Total Flavonoids Content Quantification

AEAA has a moderate level of total phenolic content (TPC) of 12.251 mg GAE/g DW. Total flavonoids, a subclass of phenolic are present in lower amounts at 3.290 mg QE/g DW. AEAA′s phenolic and flavonoid content suggests its biological potential (Table 2).

**Table 2:** Results of quantitative analysis.

	TPC (mg GAE/g DW)	TFC (mg QE/g DW)
AEAA	12.251±0.078	3.290±0.026

### 3.3 Antioxidant Activity

The antioxidant activity of AEAA is tested using five different mechanism's reaction assays. Our extract demonstrated significant antioxidant activity in all the assessed tests, which were examined against multiple standards such as BHT, EDTA, and Vitamin C. The outcomes related to the radical scavenging activity were as follows: (DPPH IC_50_=0.269±0.05, H_2_O_2_ IC_50_=0.0376±0.003 and Iron Chelation IC_50_=0.732±0.037), and the results related to the reducing power methods were as follows: (TBA %=7.843±0.522 and FTC %=24.662±0.464). The results are presented as IC_50_ and I% values, calculated through a linear regression approach and expressed as mean ± SD (n=3) (Table 3).

**Table 3:** Results of antioxidant activity.

	IC_50_ mg/ml			I%	
	DPPH	H_2_O_2_	Iron Chelation	TBA	FTC
AEAA	0.269±0.05	0.0376±0.003	0.732±0.037**	7.843±0.522*	24.662±0.464*
BHT	0.026±0.0006	NT	NT	84.848±6.053	67.645±2.913
EDTA	NT	NT	0.003±0.0005	NT	NT
Vitamin C	NT	0.0172±0.0013	NT	36.666±2.957	50.963±1.454

All values are mean of triplicate determinations. Different numbers within a column are significantly different at p <0.05 (* p <0.001, **p <0.0001). NT: Not Tested.

### 3.4 In Vitro Anti‐Inflammatory Activity

The *in vitro* anti‐inflammatory activity of AEAA was tested using two different mechanism reaction assays. Our extract demonstrated significant anti‐inflammatory activity in both methods, in the BSA assay, AEAA exhibited stronger anti‐inflammatory activity than Aspirin® with IC_50_ values of 2.56329 and 3.920135 mg/mL, respectively. However, in EA method the AEAA showed lower anti‐inflammatory activity than Aspirin®, with a much higher IC_50_ value 11.40296 mg/mL (Table 4).

**Table 4:** Results of the *in vitro* anti‐inflammatory Activity of AEAA and the Aspirin®.

Samples	IC_50_ mg/ml	
	BSA	EA
AEAA	2.56329±0.0211*	11.40296±0.0816**
Aspirin®	3.920135±0.075	3.985014±0.0209

All values are mean of triplicate determinations. Different numbers within a column are significantly different at p <0.05 (* p <0.0001, **p <0.00001).

### 3.5 Antibacterial Activity

Bacterial sensitivity to AEAA extract is determined by the diameter of the inhibitory zones (in mm). The results of AEAA antibacterial activity were examined against five bacterial strains (*E. coli*, *B. subtilis*, *K. pneumoniae*, *S. aureus*, and *P. aeruginosa*) are illustrated in the Table 5.

**Table 5:** Results of antibacterial activity of AEAA.

	E. coli	B. subtilis	K. pneumoniae	S. aureus	P. aeruginosa
D1	27±0	34±0	30.96±0.057	25±0	24.56±0.51
D2	23.86±1.8	27.86±0.23	23.2±0.17	23±0	21±0
D3	20.2±1.7	23±0	19.2±0.34	20±0	16.86±0.23
Gen	27	0	23	25	28

**D1**: 100 mg/ml; **D2**: 75 mg/ml; **D3**: 50 mg/ml

### 3.6 Acute Toxicity

In the acute toxicity testing, the oral limit dose of AEAA did not result in any deaths among the animals that were examined. During both the short‐term and the long‐term studies, there was no evidence of a fatal effect. The 14‐day trial revealed no signs of toxicity or behavioral changes in the animals, leading to the conclusion that AEAA is safe.

### 3.7 In Vivo Anti‐Inflammatory Activity

The anti‐inflammatory effect of the aqueous extract obtained by decoction of plants of *A. aristata* was assessed using mouse model carrageenan‐induced paw edema, the results represent the mean ± SEM (n=5–6 per group). AEAA exhibited significant anti‐inflammatory activity, demonstrating an inhibition value of 56.51 % (Table 6). This value is approximately 67.63 % of the anti‐inflammatory efficacy of standard Ibuprofen®, which has an inhibition value of 83.58 %. This comparison suggests that AEAA is less efficacious than Ibuprofen® in alleviating inflammation; however, it retains a significant portion of Ibuprofen's efficacy.

**Table 6:** Results of *in vivo* anti‐inflammatory activity.

Group	Weight average (g)		% Edema	ERP%
	Left paw	Right paw		
Physiological water	0.104±0.0102	0.07±0.008	43.79	/
Ibuprofen^®^	0.169±0.0162	0.139±0.0185	7.19	83.58
AEAA	0.075±0.0106	0.063±0.0137	16.21	56.51

### 3.8 In Vivo Sedative Activity

The sedative activity of AEAA was evaluated using locomotor activity. The results are presented as the mean ± SEM (n=6 per group). The sedative activity of the extract AEAA is significantly lower than that of the standard Haloperidol®, which exhibits a much stronger inhibition at 90.77 %, with an inhibition value of 48.98 %. The relative potency calculation reveals that AEAA is approximately 54 % as effective as Haloperidol^®^, and the percentage difference indicates that AEAA′s effect is approximately 46.03 % less potent than Haloperidol^®^. Although Haloperidol exhibits superior sedative effects, AEAA continues to be clinically relevant (Table 7).

**Table 7:** Results of *in vivo* sedative activity.

**Group**	**Locomotor activity in 30 min**	**MRP I%**
**Physiological water**	482.16±368.92	/
**AEAA**	246±239.13	48.98
**Haloperidol^®^**	44.5±29.81	90.77

### 3.9 Correlation Between Studied Parameters

**Figure 3:**
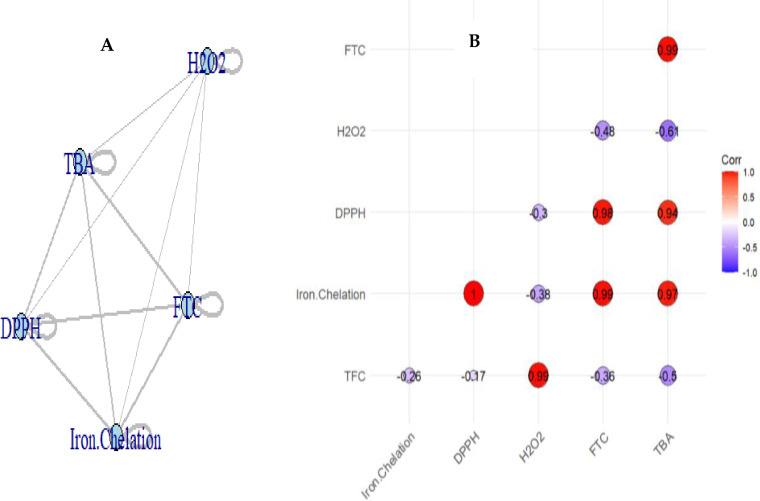
Relationships between antioxidant evaluation assays: **A**; Network graph analysis. **B**; Person heatmap correlations.

The connections between the antioxidant results indicate that the various methods of measuring antioxidants are connected and could offer complimentary insights about the antioxidant characteristics of the samples under examination. For example, the edge between DPPH and TBA indicates that the free radical scavenging ability is related to the level of lipid peroxidation. Similarly, the edge between FTC and H_2_O_2_ suggests a relationship between the ability to inhibit lipid oxidation and the capacity to scavenge hydrogen peroxide. This allowed us to choose the best assays to thoroughly assess the antioxidant capacity of different compounds or extracts by having a thorough understanding of these interactions. It can examine the intricate relationships between various antioxidant evaluation techniques and spot possible trends or correlations in the data with the help of this network graph visualization (Figure 3B). Additionally, there are somewhat favorable associations (0.4 < r <0.7) between H_2_O_2_ and DPPH and between TBA and FTC. Although the intensity of the association is not as great as in the strongly correlated pairings, these associations show that changes in one variable are linked to proportionate changes in the other. Furthermore, several negative associations are demonstrated, especially between the other factors and iron chelation. This implies that possibly as a result of antagonistic or competitive mechanisms, elevated iron chelation activity is inversely correlated with the other parameters tested (Figure 3A).

**Figure 4:**
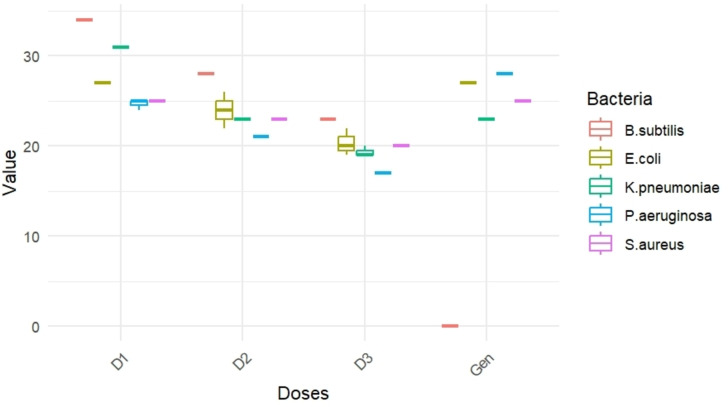
Comparative test of the antibacterial potential of *A. aristata* extract by doses.

*B. subtilis* demonstrated the highest sensitivity, recording the largest inhibition zone at 34 mm, highlighting the strong effect of AEAA extract against this Gram‐positive strain. The Gram‐negative bacteria *E. coli* and *K. pneumoniae* also exhibiting substantial sensitivity, marked by inhibition zones of 27 mm and 30.96±0,057 mm in **D1** (100 mg/ml) respectively. The **D2** (75 mg/ml) showed reduced effectiveness across all bacteria, although *B. subtilis* remained the most sensitive. **D3** exhibited the lowest activity levels, marking it as the least effective dosage. In the general activity Gentamicin (Gen), *P. aeruginosa*, another Gram‐negative bacterium, displayed the highest inhibition zone at 28 mm, while *B. subtilis* showed no response. Gen and the AEAA in **D1** (100 mg/ml), have the same inhibitory effect on the bacteria tested *E. coli* and *S. aureus*, with average diameters of inhibitory zone of 27 and 25 mm respectively (Figure 4).

**Figure 5:**
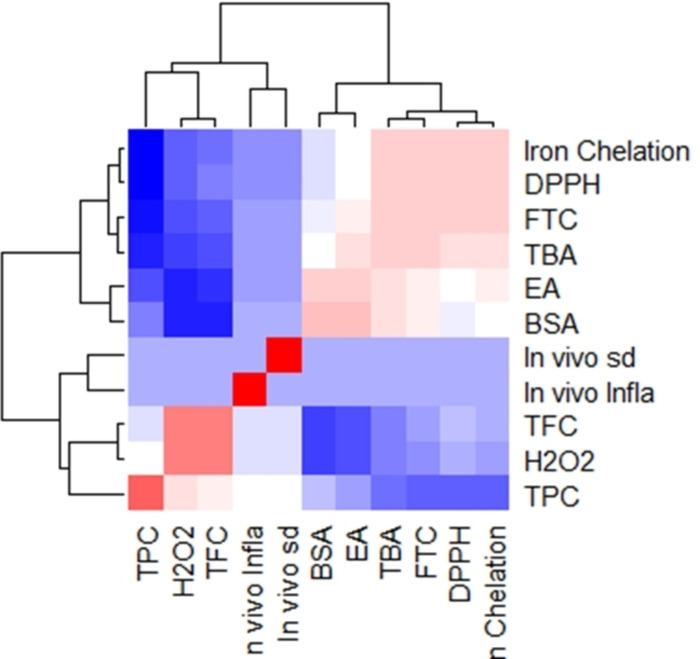
Correlation heatmap of experimental parameters. Where, total phenolic (TPC) total flavonoids (TFC) *in vitro* (BSA and EA) and *in vivo* (in vivo Infla) anti‐inflammatory and the sedative (in vivo sd).

The heatmap correlations between the amount of total phenolic TPC and TFC and the tested antioxidant, *in vitro* anti‐inflammatory (BSA and EA) and in in vivo anti‐inflammatory and the sedative activities demonstrates high positive correlations (r >0.7) between the sedative and the anti‐inflammatory actions, as well as between EA and BSA denaturation. These results indicate a close association between these characteristics and the possibility of shared underlying mechanistic linkages (Figure 5).

## 4 Discussion

The phytochemical compounds of AEAA were detected using UPLC‐ESI‐MS analysis by comparing the retention times with those of standards in both positive and negative modes, revealed the presence of variety phytochemicals, notably phenolic acids, flavonoids, and other important secondary metabolites. Significant phenolic acids were detected in AEAA including 2‐methoxybenzoic acid, p‐Anisic acid, coumaric acid, ferulic acid, cinnamic acid, and chlorogenic acid. Phenolic acids are found in extremely high concentrations across the plant kingdom, which enhances the pharmacological effects of medicinal plants.^[30]^ Many medicinal plants contain vitexin, a C‐glycosylated flavonoid, and myricetin, an O‐glycosylated flavonoid. These natural chemicals were detected in AEAA and exhibit a multitude of biological activities and hold substantial potential for research and development.[^31^, ^32^] Additionally, coumarin derivative esculin hydrate and a monoterpene phenol thymol were detected in AEAA. The phytochemicals support the plant‘s traditional medical uses and provide a solid foundation for further pharmacological research, highlighting its potential to help with inflammation, oxidative stress, and microbial infections.[^33^, ^34^, ^35^]

To the best of our knowledge, this study is the first to explore the antioxidant activities of the aqueous extract of the plant *A. aristata*. Currently, there are no published studies that specifically examine this extract using the antioxidant tests outlined in Table 3. In this study the antioxidant capabilities of AEAA were assessed through five distinct antioxidant assays: DPPH, H_2_O_2_, Iron Chelation, TBA, and FTC tests, which encompass diverse reaction processes and circumstances. The antioxidant activities tested are classified into radical scavenging activity, including DPPH assay, hydrogen peroxide radical scavenging, and iron chelation assay, which measure the extract‘s ability to neutralize free radicals or prevent radical formation. The reducing power methods include the TBA and FTC assays, assessing the extract's effectiveness in inhibiting lipid peroxidation and reducing peroxides, where the FTC test measures the production of peroxide compounds during the initial stage of oxidation, while the TBA test measures the secondary product of oxidation. Peroxides undergo gradual degradation into compounds with reduced molecular weights during the oxidation process, malonaldehyde is an example of such a compound and is quantified using the TBA method.[^36^, ^37^] According to Kasote et al., 2015 and El‐Nashar et al., 2022 researches have shown that plants have the natural potential to biosynthesize a wide variety of non‐enzymatic antioxidants, which have the ability to reduce the oxidative damage that is caused by reactive oxygen species (ROS).[^38^, ^39^]

In the present study, our extract exhibited strong antioxidant activity against the free radicals DPPH and hydrogen peroxide with IC_50_ values of 0.269±0.05 and 0.0376±0.003 mg/mL, respectively. In addition, AEAA showed best chelation of the iron metal with IC_50_ value of 0.732±0.037 mg/mL. Whereas, the antioxidant activity related to the reducing power methods the AEAA revels very low inhibition percentage with I%=24.662±0.464 in the FTC method and I%=7.843±0.522 in the TBA method when compared to standards vitamin C and BHT. This gap is associated with the types and amounts of bioactive chemicals, including polyphenols and flavonoids and their mechanism of action.[^40^, ^41^] Our results are in accord of those of our published papers, where the crude extract of the plant *A. aristata* exhibited a potent antioxidant activity stronger than the standard ascorbic acid against the free radicals DPPH and ABTS with values of IC_50_=0.040 ±0.003 mg/mL and IC_50_=0.005 ±5.77×10^−5^ mg/mL ABTS, respectively. Moreover, the ethyl acetate extract of the plant *A. aristata* displayed high antioxidant capacity with IC_50_=0.097±0.003 mg/mL in DPPH assay and IC_50_=0.077±0.003 mg/mL in the ABTS assay, these results support our findings and confirm the strong free radicals scavenging capacity of this plant.[^14^, ^15^]

Based on our findings, it appears that the antioxidant characteristics of AEAA can be related to phenolic and flavonoid molecules that contain numerous hydroxyl groups and demonstrate a high level of antioxidant activity.^[42]^ In addition, the results of UPLC/MS‐MS analysis revealed that the health advantages of polyphenols are mostly attributable to their antioxidant activity. This activity is accomplished by scavenging free radicals through the use of hydroxyl groups via hydrogen‐atom, electron transfer and/or metal ion chelation.^[43]^ An abundance and variety of bioactive chemicals in the plant *A. aristata* substantially augment its antioxidant activities, rendering it a potent natural antioxidant source. These chemicals function synergistically to neutralize free radicals and absorb oxygen radicals, thereby enhancing the overall antioxidant capacity of the plant. The presence of several antioxidants offers a more thorough defense against oxidative stress, as distinct substances may address multiple pathways and mechanisms for free radical generation and propagation. The antioxidant activity of the AEAA can be principally attributable to the presence of chlorogenic acid, caffeic acid, and ferulic acid. Chlorogenic acid gets rid of ROS by giving up hydrogen atoms, staying stable as phenoxy radicals and binding to iron ion. Nrf2 upregulation also raises the levels of antioxidant enzymes like glutathione peroxidase, superoxide dismutase, and catalase. These enzymes help keep the balance of redox reactions in cells. In addition, it safeguards DNA and lipids from oxidative damage and controls stress‐related pathways like NF‐κB.^[44]^ Caffeic acid absorbs ROS, prevents lipid peroxidation, and shields DNA from UV−B damage. The conjugated structure stabilizes radicals, boosting its antioxidant activity, although its dual role as an antioxidant and prooxidant depending on dose complicates it.^[45]^ By getting rid of ROS, stopping oxidative enzymes, chelating transition metals, stabilizing free radicals by resonance, and absorbing UV light, ferulic acid keeps cells safe from oxidative damage.^[46]^ All of these chemicals boost the AEAA antioxidant capability, protecting it from oxidative damage.

Our antibacterial activity results of AEAA show variability in bacterial type and dose responses under different conditions. Doses had a substantial impact in most cases, while a significant effect of bacterial type appeared in only one experiment. The results of antibacterial activity achieved by Rahman et al., 2011, by testing the methanolic extract of the plant *Atractylis carduus* presented the high activities in gram positive bacteria *B. cereus* and *S. aureus* marked by inhibition zones of 40 mm and 33 mm in (23 mg/ml) respectively,^[47]^ these inhibition zones are much closed to our findings. Nerveless, the petroleum ether, ethyl acetate and n‐butanol extracts of the plant *Atractylis cancellata* inhibited the growth of all the tested bacteria *S. aureus*, *P. aeruginosa*, *Citrobacter koseri and Proteus mirabilis* by very low inhibition zones of 12±0.1 mm at maximum in a dose of 100 ug/ml.^[48]^ The exhibition of antibacterial efficacy against both Gram‐positive and Gram‐negative bacteria may suggest the existence of broad‐spectrum antibiotic agents.^[49]^ Several studies have focused on a particular medicinal application of Cinnamic acid and Curcumin molecules as antifungal, antimicrobial including *Staphylococcus aureus* and *Escherichia coli*.[^50^, ^51^, ^52^] The antibacterial activity of AEAA could be attributed to the presence of these compounds in our extract that mentioned in the UPLC‐ESI‐MS‐MS analysis part. Some microbes have heightened sensitivity to particular groups of chemicals. For example, fungi exhibit greater susceptibility to cinnamic aldehydes, whereas cinnamic acids, esters, and amides generally exert a more pronounced effect on bacteria.^[53]^

Protein denaturation is a critical element in inflammation, as evidenced by credible research.[^54^, ^55^] Researchers frequently conduct denaturation studies using egg albumin and bovine serum albumin, recognizing the connection between protein denaturation in tissues and the onset of inflammatory and arthritic disorders, which is associated with the production of autoantigens. The capacity to prevent albumin denaturation under thermal exposure at physiological pH is a defining trait of non‐steroidal anti‐inflammatory medicines.^[55]^ Protein denaturation is caused by changes in disulfide bonds, hydrogen bonds, hydrophobic interactions, and electrostatic forces.^[56]^ In the present study, the aqueous extract of *A. aristata* exhibited strong anti‐inflammatory activity higher than the Aspirin® in the BSA denaturation method with IC_50_ values of 2.56329 mg/mL and 3.920135 mg/mL, respectively. In the EA denaturation method AEAA also showed good an anti‐inflammatory with IC_50_ values of 11.40296 mg/mL. The results indicate the strong anti‐inflammatory activity of AEAA in both methods. These results are in accord with those of our published paper using experimental crude extract of the plant *A. aristata* and the standard diclofenac sodium, which exhibited a concentration‐dependent inhibition of BSA denaturation with inhibition of 70.84±0.10 mg/mL at concentration of 1.5 mg/mL.^[14]^

The carrageenan‐induced paw edema model is a significant approach for assessing the *in vivo* anti‐inflammatory efficacy of experimental extracts. In the present study AAEA demonstrated anti‐inflammatory action, with an inhibition value of 56.51 % (Table 6). This figure constitutes roughly 67.63 % of the anti‐inflammatory efficacy of Ibuprofen®, which possesses an inhibition value of 83.58 %. This comparison indicates that AEAA is less effective than Ibuprofen in reducing inflammation; yet, it preserves a substantial degree of Ibuprofen's potency. By comparing this finding with those of our published study by the crude extract of the plant *A. aristata* using the same method, which exhibited an inhibition percentage equal to 62.98 % at the same dose, the AEAA showed a slightly less ant‐inflammatory activity than the crude extract of this plant.^[14]^ Many findings of anti‐inflammatory conducted on species of Atractylis genus are in close correlation with experimental studies of species with bioactive potential and their main compounds; their confirmed activities include, among others, flavonoids, which constitute an important group of the main compounds in species like *A. flava*,^[57]^ *A. gummifera* ^[58]^ and *A. cancellata* ^[48]^ that show anti‐inflammatory activities. Some studies have linked the anti‐inflammatory action of different compounds to the suppression of certain signaling pathways or the inhibition of the release of several pro‐inflammatory cytokines. Prior research indicated a potent *in vitro* and *in vivo* potential of secondary metabolites such as phenolic acids, flavonoids, flavonols, and saponins due to their erythrocyte membrane stabilizing properties and high affinity for cation binding. Many Compounds in the Atractylis genus have anti‐inflammatory properties that may be primarily explained by their regulation of pro‐inflammatory cytokines or signaling pathways linked to inflammation.[^59^, ^60^, ^61^]

The majority of sedative‐hypnotic agents in clinical use today are synthetic compounds, classified primarily into three categories: barbiturates, benzodiazepines, and newer non‐benzodiazepine sedative‐hypnotics. While these drugs effectively induce sedative‐hypnotic effects, they are also associated with a range of undesirable side effects, such as headaches, dizziness, excessive drowsiness, rebound insomnia, respiratory depression, and potential drug interactions. These adverse reactions have prompted significant research into alternative natural products that may offer sedative‐hypnotic properties without the drawbacks commonly observed with synthetic options.^[62]^ The sedative activity of the AEAA extract was evaluated and compared with the standard drug Haloperidol® by measuring *in vivo* locomotor activity. Data indicated that the control group exhibited an average locomotor activity of 482.16±3.92, reflecting the normal activity level without any sedative influence. In contrast, the AEAA group showed a reduction in locomotor activity to 246±2.13, representing a 48.98 % decrease compared to the control group. This reduction suggests a moderate sedative effect exerted by the AEAA extract. The Haloperidol® group, however, demonstrated a sharp decrease in locomotor activity, recording an average of 44.5±0.81 at dose of 2 g/kg with a 90.77 % reduction relative to the control, indicating a strong sedative effect of Haloperidol® that significantly surpasses the sedative action of AEAA. The sedative activity of our extract showed slightly less activity than the crude extract of this plant with inhibition of 52.12 % at the same concentration.^[14]^

The plant *A. aristata* represents a notable example of a natural remedy traditionally employed to address conditions such as dizziness and headaches.^[63]^ In addition to its well‐documented calming effects, this plant holds promise as a safer alternative to synthetic sedative‐hypnotics, which are often accompanied by a range of undesirable side effects. Due to its intrinsic therapeutic properties, *A. aristata* has the potential to mitigate the adverse reactions commonly observed with manufactured drugs, while providing comparable benefits in terms of sedation and symptom relief. This suggests that the plant *A. aristata* could offer an effective, side‐effect‐free option for individuals seeking natural treatments for conditions typically managed by synthetic sedatives. The results suggest that AEAA extract possesses sedative properties, albeit to a lesser extent than Haloperidol®. This positions AEAA as a potential natural agent for sedation, warranting further studies to elucidate its mechanism of action and to optimize dosing. Always and as known the biological activities of the medicinal plant attributed to their phytochemical constituents for that the sedative effect of our extract could be directly correlated with the compounds mentioned in the results of UPLC‐ESI‐MS‐MS. In a study conducted by Hartley and McLachlan (2022) indicated that the thymol compound has an effect on the central nervous system, where it acts as an agonist for GABA (gamma‐aminobutyric acid) receptors. These receptors are known to calm neural activity and reduce anxiety. Thymol's inhibition of hyperactivity may contribute to its anti‐anxiety effects ^64]^. In addition, other study conducted by reported that the coumaric acid and curcumin as an antioxidant, reducing oxidative stress in the brain, which is linked to increased anxiety and stress. Its anti‐anxiety effect is enhanced by reducing oxidative stress in the central nervous system.[^65^, ^66^]

## 5 Conclusions

In summary, aqueous extract of the plant *A. aristata* has demonstrated considerable promise as a multifaceted therapeutic agent, exhibiting potent antioxidant, anti‐inflammatory, antibacterial, and sedative activities in both *in vitro* and *in vivo* models. These findings underscore AEAA's ability to influence critical biological pathways, suggesting its potential in the management of oxidative stress‐related disorders, inflammatory conditions, and microbial infections. Moving forward, it is imperative to investigate the molecular mechanisms driving these biological effects to better understand its therapeutic potential. Additionally, clinical trials are necessary to establish the safety, efficacy, and pharmacokinetic profile of AEAA in humans, while research into the development of optimized delivery systems will be crucial to enhance its bioavailability and therapeutic efficacy. Further exploration of its interactions with other natural compounds, as well as long‐term toxicity studies, will be vital in assessing its suitability for clinical use. To ensure the widespread applicability of AEAA, the development of sustainable extraction techniques and large‐scale production methods should be prioritized. These efforts will facilitate the integration of AEAA into natural‐based therapeutic strategies, solidifying its place as a promising candidate for future medical applications.

## Conflict of Interests

The authors declare no conflicts of interest.

## Data Availability

The data that support the findings of this study are available from the corresponding author upon reasonable request.
